# Genome–wide binding of transcription factor ZEB1 in triple‐negative breast cancer cells

**DOI:** 10.1002/jcp.26634

**Published:** 2018-05-10

**Authors:** Varun Maturi, Stefan Enroth, Carl‐Henrik Heldin, Aristidis Moustakas

**Affiliations:** ^1^ Department of Medical Biochemistry and Microbiology, Science for Life Laboratory, and Ludwig Institute for Cancer Research Uppsala University Uppsala Sweden; ^2^ Department of Immunology, Genetics and Pathology, Science for Life Laboratory Uppsala University Uppsala Sweden

**Keywords:** EMT, TGFβ, TIMP, ZEB1

## Abstract

Zinc finger E‐box binding homeobox 1 (ZEB1) is a transcriptional regulator involved in embryonic development and cancer progression. ZEB1 induces epithelial‐mesenchymal transition (EMT). Triple–negative human breast cancers express high *ZEB1* mRNA levels and exhibit features of EMT. In the human triple–negative breast cancer cell model Hs578T, ZEB1 associates with almost 2,000 genes, representing many cellular functions, including cell polarity regulation (DLG2 and FAT3). By introducing a CRISPR‐Cas9‐mediated 30 bp deletion into the *ZEB*1 second exon, we observed reduced migratory and anchorage‐independent growth capacity of these tumor cells. Transcriptomic analysis of control and ZEB1 knockout cells, revealed 1,372 differentially expressed genes. The *TIMP metallopeptidase inhibitor* 3 and the *teneurin transmembrane protein* 2 genes showed increased expression upon loss of ZEB1, possibly mediating pro‐tumorigenic actions of ZEB1. This work provides a resource for regulators of cancer progression that function under the transcriptional control of ZEB1. The data confirm that removing a single EMT transcription factor, such as ZEB1, is not sufficient for reverting the triple–negative mesenchymal breast cancer cells into more differentiated, epithelial‐like clones, but can reduce tumorigenic potential, suggesting that not all pro‐tumorigenic actions of ZEB1 are linked to the EMT.

**Abbreviations**
DLG: discs large MAGUK scaffold protein
EMT: epithelial mesenchymal transition
FAT: FAT atypical cadherin
TGFβ: transforming growth factor β
TENM: teneurin
TIMP: tissue inhibitor of metallopropoteinases
ZEB: zinc finger E‐box binding homeobox 1

## 1 INTRODUCTION

An important and not fully understood aspect of cancer development is the relationship between differentiation and invasive capacity developed by tumor cells, which promotes metastasis (Lambert, Pattabiraman, & Weinberg, 2017; Nguyen, Bos, & Massagué, 2009). Epithelial tumors present morphogenetic plasticity that allows de‐differentiation and re‐differentiation to take place during their evolution; the epithelial‐mesenchymal transition (EMT) represents such tumor cell plasticity (Nieto, Huang, Jackson, & Thiery, 2016). EMT empowers carcinomas with stem cell features, and facilitates remodeling of junctional complexes with subordinate cytoskeletal adaptation, preparing tumor cells for invasion into their microenvironment (Nieto et al., 2016). A hallmark of EMT is the degradation of the junctional transmembrane protein E‐cadherin and the transcriptional repression, and silencing via methylation, of the corresponding *CDH1* gene (Berx & van Roy, 2009).

In carcinomas, but also during embryogenesis, EMT is guided by extracellular growth factors, such as transforming growth factor β (TGFβ), hepatocyte growth factor, fibroblast growth factor (FGF), and the Notch receptor system (Nieto et al., 2016). The transmembrane TGFβ receptors type II and type I, members of the receptor serine/threonine kinase family, that also exhibit weak tyrosine kinase activity, signal via Smad proteins, lipid, and protein kinases and control gene expression via specific transcription factors (Moustakas & Heldin, 2012). TGFβ contributes to metastatic progression of carcinomas, by promoting EMT, suppressing anti‐tumoral immune responses, and by enhancing the differentiation of cancer‐associated fibroblasts and the growth of the tumor vasculature (Bierie & Moses, 2006).

A key mechanism by which TGFβ initiates and propagates EMT involves the transcriptional regulation of specific EMT transcription factors (EMT‐TFs) (Moustakas & Heldin, 2012). The EMT‐TFs include zinc finger proteins (Snail1, Snail2/Slug), zinc finger, and homeobox domain proteins (zinc finger E‐box binding homeobox 1, ZEB1/ZFHX1A/δEF1 and ZEB2/SIP1), and basic helix loop helix proteins (E47, Twist1) (Nieto et al., 2016). For example, TGFβ signaling induces the expression of the high mobility group A2 (HMGA2) chromatin factor, which induces *Snail1* and *Twist1* expression and together, HMGA2, Snail1, and Twist1 repress and recruit DNA methyltransferases to the *CDH1* gene (Tan et al., 2015). Furthermore, Snail1 and Twist1 cooperatively induce ZEB1 in response to TGFβ (Dave et al., 2011).

Thus, ZEB1 is best known as a transcriptional repressor of *CDH1* and inducer of EMT in breast and other carcinomas (Eger et al., 2005). During embryogenesis, ZEB1 controls several mesenchymal cell lineages giving birth to cranial, limb, thoracic, and vertebral bones and cartilage (Takagi, Moribe, Kondoh, & Higashi, 1998). For this reason, mice lacking ZEB1 die early after birth due to skeletal and thymic defects (Takagi et al., 1998). In mediating EMT, ZEB1 represses epithelial polarity genes, such as *Crumbs3* and *Lgl2* (Aigner et al., 2007; Spaderna et al., 2008). Repression of laminin‐332 (*LAMC2*) and integrin‐β4 (*ITGB4*) genes contributes to the invasiveness associated with EMT in prostate carcinoma cells (Drake et al., 2010). Extensive alternative splicing occurs during EMT, in part mediated by ZEB1, which transcriptionally represses the epithelial splicing regulatory protein genes, favoring expression of spliced isoforms of the FGF receptors that help maintain EMT in breast cancer cells in response to TGFβ (Horiguchi et al., 2012). The micro‐RNA 200 (*miR‐200*) gene family is actively repressed by ZEB1 in response to TGFβ signaling; *miR‐200* pairs with the *ZEB1* and *TGFβ2* mRNAs and inhibits their translation, thus forming a double‐negative feedback loop that is critical for breast carcinoma EMT (Burk et al., 2008). Epithelial *miR‐200* expression is maintained by the transcription factor c‐Myb, which is transcriptionally repressed by ZEB1 (Hugo et al., 2013; Pieraccioli, Imbastari, Antonov, Melino, & Raschella, 2013). Thus, ZEB1 represses several genes in carcinomas, but also activates transcription, when pairing with the co‐activator YAP of the Hippo pathway, inducing mesenchymal gene expression (Lehmann et al., 2016).

ZEB1 promotes metastasis in breast and pancreatic carcinomas (Krebs et al., 2017; Spaderna et al., 2008). For example, ZEB1 facilitates bone‐specific metastasis of breast carcinomas by inducing expression of noggin, follistatin and chordin‐like 1, extracellular antagonists that inactivate ligands of the activin, and bone morphogenetic protein branches of the TGFβ family (Mock et al., 2015). ZEB1 contributes to the resistance to anti‐cancer therapy by establishing a repressive chromatin state (Meidhof et al., 2015). Resistance also extends to radiotherapy, as radiation stabilizes ZEB1 and promotes signaling by the CHK1 protein kinase, stimulating homologous DNA recombination (Zhang et al., 2014).

Overall, the transcription factor ZEB1 mediates functions that link cancer EMT to TGFβ signaling, metastatic dissemination, stemness, and resistance to therapy. This generates a strong interest in deciphering the complete regulatory network downstream of ZEB1 in carcinomas. Based on this premise, we analyzed the genome–wide association of ZEB1 and evaluated the loss of function mutation in ZEB1 in breast carcinomas.

## 2 MATERIALS AND METHODS

### 2.1 Cell and CRISPR cas9 knockout models

Hs578T and MDA‐MB‐231 cells were cultured in Dulbecco's modified Eagle's medium (DMEM) and T47D cells in Roswell Park Memorial Institute (RPMI)‐1640 supplemented with 10% fetal bovine serum (FBS) in the presence of penicillin‐streptomycin. Cells starved for 18 hr in serum‐free DMEM or RPMI were stimulated with 5 ng/ml TGFβ1 (recombinant human TGFβ1, PeproTech Nordic, Stockholm, Sweden). TGFβ receptor type I kinase inhibitor GW6604 (dissolved in dimethylsulfoxide [DMSO]) was synthesized by the Ludwig Cancer Research Ltd. Hs578T cells were transfected with CRISPR Cas9 and HDR plasmids targeting ZEB1, obtained from Santa Cruz Biotech Inc., Santa Cruz, CA. After 2 days of post‐transfection, cells were selected with puromycin; single cell colonies were subcultured. Knockout clones were validated using immunoblotting and mutated DNA sequences were analyzed after conventional PCR.

### 2.2 Soft agar colony formation assay

Six‐well plates were plated with 1% noble agar in DMEM/10% FBS (Borowicz et al., 2014). Cells (5,000/well) were resuspended in 0.5% noble agar in DMEM/10% FBS, and layered on top of the agar. Solidified plates were then incubated at 37 °C for 16 days, cells were viewed under a phase–contrast microscope, and colonies were counted. Each experiment was performed three times and each condition included triplicates.

### 2.3 T‐Scratch assay

Cells were seeded in a six‐well plate such that they were 90% confluent the following day. A “ **+ **” scratch was made on the cell layer using a pipetman tip. Cells were washed with PBS twice and left in DMEM. The scratched area was photographed under a phase contrast microscope. The culture was left at 37 °C overnight and then photographed under the same microscope. Cell images on day 1 and 2 were analyzed using the T‐Scratch software (http://www.cse-lab.ethz.ch/index.php?& option = com_content&view = article&id = 363) to quantify cell migration. Each experiment was performed three times and each condition included triplicates.

### 2.4 Immunoblotting

Total proteins from Hs578T wild‐type and ZEB1 knockout clones were extracted in nonidet P‐40 (NP‐40) containing lysis buffer (20 mM Tris‐HCl [pH 8.0], 1% NP‐40, 150 mM NaCl, 2 mM EDTA, complete protease inhibitor cocktail‐Roche Diagnostics Scandinavia AB, Bromma, Sweden). Lysates were heated at 95 °C for 5 min and subjected to SDS‐PAGE.

### 2.5 Antibodies

The following antibodies were used: rabbit polyclonal anti‐ZEB1, rabbit polyclonal anti‐Slug, monoclonal rabbit anti‐HMGA2, monoclonal mouse anti‐β‐actin (Santa Cruz Biotech, Inc., Sweden); rabbit polyclonal anti‐ZEB1 (Novus Biological, Littleton, CO); rabbit monoclonal anti‐Snai1 (Cell Signaling Technology, Leiden, The Netherlands); rabbit control IgG‐ChIP grade and mouse control IgG‐ChIP grade (Abcam, Cambridge, United Kingdom); monoclonal mouse anti‐CDH11 (Thermo Fisher Scientific, Waltham, MA); polyclonal rabbit anti‐N‐Cadherin, monoclonal mouse anti‐PAI1 (BD Transduction Laboratories AB, Stockholm, Sweden); monoclonal rabbit anti‐fibronectin (Sigma–Aldrich, Stockholm, Sweden); rabbit polyclonal anti‐pSmad2 was made in house (Souchelnytskyi et al., 1997); rabbit polyclonal anti‐CAR was a gift of Jonas Fuxe, Karolinska Institute, Sweden.

### 2.6 Immunofluorescence microscopy

Cells were cultured on glass slides in six‐well plates with DMEM/10% FBS, fixed in 3.7% paraformaldehyde for 10 min at room temperature, washed twice with PBS and permeabilized in 1% Triton X–100 in PBS for 15 min at room temperature. Cells were washed in PBS‐T (0.2% Triton X–100 in PBS) and left for blocking in 20% FBS in PBS‐T for 1 hr at room temperature. Cells were incubated overnight with primary antibodies diluted in blocking solution (1:200). Cells were washed three times with PBS‐T, incubated with secondary antibody diluted with blocking solution (1:500) for 1 hr at room temperature, washed with PBS‐T three times and incubated with TRITC‐conjugated phalloidin (1:1,000 dilution in PBS; Sigma–Aldrich AB, Stockholm, Sweden) for 15 min at room temperature in the dark. After washing with PBS twice and mounting with Fluoromount G (SouthernBiotech, AH Diagnostics, Solna, Sweden) containing 4',6‐diamidino‐2‐phenylindole (DAPI, Sigma–Aldrich AB, Stockholm, Sweden), images were obtained on a ZEISS axio‐imager M2 fluorescence microscope and the Zen software.

### 2.7 Chromatin immunoprecipitation

Cells were cultured and fixed with 2% formaldehyde for 10 min at 37 °C, washed in ice‐cold PBS twice, scraped in PBS and spun down at 4,000 rpm for 5 min. Cell pellets were lysed in 1% SDS, 10 mM EDTA, 50 mM Tris, pH 8.1, with protease inhibitors for 20 min on ice, then sonicated to an average DNA fragment size of 250 bp. Input chromatin aliquots (10%) were frozen at −20 °C. The remaining lysate was diluted 10 times in 0.01% SDS, 1.0% Triton X–100, 1.2 mM EDTA, 16.7 mM Tris‐HCl pH 8.1, 167 mM NaCl, with protease inhibitors, and proceeded for immunoprecipitation using anti‐ZEB1 or control rabbit antiserum, overnight at 4 °C. Protein‐A dynabeads were added and incubated for 2 hr at 4 °C, washed once with low salt buffer (0.1% SDS, 1% Triton X–100, 2 mM EDTA, 20 mM Tris‐HCl, pH 8.1, 150 mM NaCl), once with high salt buffer (0.1% SDS, 1% Triton X–100, 2 mM EDTA, 20 mM Tris‐HCl pH 8.1, 500 mM NaCl), once with lithium chloride wash buffer (0.25 M LiCl, 1% IGEPAL, 1% deoxycholic acid, 1 mM EDTA, 10 mM Tris‐HCl, pH 8.1), and twice with TE buffer (10 mM Tris‐HCl, pH 8.0, 1 mM EDTA pH 8.0). Beads and input samples were re‐suspended in elution buffer (1% SDS, 0.1% of 1M NaHCO_3_) and mixed up and down for 30 min and left for de‐crosslinking in the presence of NaCl at 65 °C overnight. The chromatin was subjected to proteinase‐K digestion followed by phenol‐chloroform extraction. Respective input was used to normalize the DNA in each sample that was subjected to ChIP. The extracted DNA was subjected to either PCR of sequencing analysis. The ChIP‐qPCR primers were: human *CDH1*, forward 5′‐GGCCCTGCAGTTCCTTGGCT‐3′, reverse 5′‐AGTGAGCAGCGCAGAGGCTG‐3′; human *DLG2*, forward 5′‐CCTGCATCCATGTTGCCAC‐3′, reverse 5′‐ AACCCAGGTGCCCATTAGTG‐3′; human *FAT3* forward 5′‐GATTTGCCACAGAGAGCAGC‐3′, reverse 5′‐TCCCTTCACTTCTAAGCCATCT‐3′; human *TENM2* forward 5′‐ TGCAAAGAGGCCACGATTCT‐3′, reverse 5′‐CTGAGCCGTGTTTGCCATTG‐3′; human *TIMP3* forward 5′‐ TGGCTATGTTGAGACGCAAGT‐3′, reverse 5′‐ATGGCCCCTAAATCTTCAACTCA‐3′.

### 2.8 DNA library preparation and sequencing protocols

ChIP DNA was obtained with four biological and technical replicates and pooled for sequencing. ChIP DNA quality was analyzed with a Bioanalyzer. Input and ChIP DNA was sheared with a Covaris S2 sonicator (Covaris, Inc., Woburn, MA). DNA libraries were constructed using the AB Library Builder System (Life Technologies, Carlsbad, CA), followed by amplification and wildfire conversion according to the manufacturer's protocols. Library preparation was performed using the library kit (5,500 SOLiD Library Builder Fragment Core Kit + 5500W Conversion Primer Kit), after which sequencing was performed at 75 bp read length on the SOLiD 5,500W system (Life Technologies) at sequencing unit (SOLiD 5,500W FlowChip). Raw sequences were aligned to the human genome hg19 using maximum stringency with default settings via LifeScope (version 2.1 Thermo Fisher Scientific), retaining only uniquely mapped reads and unique sequences were retained in the . BAM file format.

### 2.9 Real time RT‐PCR analysis

RNA from Hs578T wild‐type and *ZEB1* knockout clones was extracted using the TRIzol reagent protocol (Ambion, LifeTechnologies, Thermo Fisher Scientific). cDNA was synthesized using the iScript synthesis kit (Bio‐Rad Laboratories AB, Solna, Sweden). Real‐time PCR was done using iTaq SYBR green supermix with ROX (Techtum Lab AB, Nacka, Sweden) using denaturation at 95 °C for 30 s, annealing at 56 °C for 30 s. and amplification at 72 °C for 45 s, repeating this for 39 cycles; a melting curve was plotted using 0.5 °C raise for every 5 s from 65 °C to 95 °C. The primers used were: human *DLG1* forward,5′‐CCAGAGGAGCAGCTGTTGAAA‐3′, reverse 5′‐GGCTTCTTCTATCTTCTGCTCACAAC‐3′; *FAT3* forward, 5′‐TCAGATCCAGGCTGAAGATCCT‐3′, reverse 5′‐GCCTGCTGTTCTCGATCCAATT‐3′; *TIMP3* forward 5′‐ CTTCGGCACGCTGGTCTA‐3′, reverse 5′‐CTGTCAGCAGGTACTGGTACTT‐3′; *TENM2* forward 5′‐GGACCTCCCCAGACTATACCAT‐3′, reverse 5′‐CAAACATCACAAGCCAGCTTTTCA‐3′; human HPRT1 forward, 5′‐GCTTCCTCCTCCTGAGCAGTC‐3′, reverse, 5′‐CACTAATCACGACGCCAGGGCTGC‐3′.

### 2.10 AmpliSeq transcriptome analysis

RNA for AmpliSeq was extracted with three biological replicates and three technical replicates. Total RNA (50 ng) was reverse‐transcribed to cDNA using Ion AmpliSeq™ Transcriptome Human Gene Expression Kit Preparation protocol (Revision A.0, Life Technologies). The acquired cDNA was amplified using Ion AmpliSeq™ Transcriptome Human Gene Expression core panel (Life Technologies) and the primer sequences were partially digested. Adaptors (Ion P1 Adapter and Ion Xpress™ Barcode Adapter, Life Technologies) were ligated to the amplicons. Adaptor‐liga**t**ed amplicons were purified using Agencourt® AMPure® XP reagent (Beckman, Coulter Inc., Brea, CA) and eluted in amplification mix (Platinum® PCR SuperMix High Fidelity and Library Amplification Primer Mix, Life Technologies) and amplified. Size‐selection and purification was conducted using Agencourt® AMPure® XP reagent (Beckman, Coulter). The amplicons were quantified using the Fragment Analyzer^™^ instrument (Advanced Analytical Technologies, INC., Ankeny, IA) with DNF‐474 High Sensitivity NGS Fragment Analysis Kit (Advanced Analytical Technologies, INC). Samples were then pooled (six or less per pool), followed by emulsion PCR on either the Ion OneTouch™ two System using the Ion PI™ Hi‐Q™ OT2 Kit (Life Technologies), or on the Ion Chef™ System using the Ion PI Hi‐Q Chef Kit (Life Technologies). The pooled samples were loaded on Ion PI™ v3 chips and sequenced on the Ion Proton™ System using the Ion PI™ Hi‐Q Sequencing 200 Kit chemistry (200 bp read length, Life Technologies). Acquired reads were aligned to the hg19 AmpliSeq Transcriptome ERCC v1 using the Torrent Mapping and Alignment Program (tmap) with default settings. Differentially expressed genes were called requiring a mean log_2_‐fold change over the replicates of two and a *q*‐value <0.05 (one‐sided *t*‐test with False Discovery Rate (FDR) adjustment).

### 2.11 Bioinformatic analysis methods

#### 2.11.1 Transcriptomic analysis in the GOBO database

The gene expression data sets for *ZEB1*, *ZEB2*, and *TIMP3* in human breast cancer cells were obtained by utilizing the cell line module of the web‐based tool gene expression‐based Outcome for breast cancer Online (GOBO) (Ringner, Fredlund, Hakkinen, Borg, & Staaf, 2011). Data were obtained using default settings and protocols prescribed by the authors.

#### 2.11.2 ChIP–Seq analysis

Aligned reads were filtered on mapping quality using samtools (Li et al., 2009) with “–q 20.” Peaks were called from ChIPs with the Input as background using MACS software (version 1.4.2) (Zhang et al., 2008) with the following changes to default settings: “–nomodel−shiftsize = 125–keep‐dup = 1.” Identified peaks were annotated to the closest gene using BEDTools (Quinlan & Hall, 2010) and protein coding genes from the refseq‐databases (hg19). FASTA‐sequence (Human Genome version GRCh37.57) +/ − 125 bp from the center of each peak‐summit as predicted by MACS was extracted and enriched DNA‐motifs were identified using TOMTOM motif identification suit (Bailey et al., 2009). Data was obtained using default settings and prescribed protocols by the authors.

#### 2.11.3 Visualization of peaks

ChIP–Seq peaks were uploaded into the UCSC genome browser (Kent et al., 2002) using custom bigwig tracks and overlayed using publicly available H3K27AC data.

#### 2.11.4 GeneOntology

Differentially expressed genes and binding targets of ZEB1 were classified into groups of molecular, biological, and cellular components using GO Panther online tool (Mi, Muruganujan, & Thomas, 2013). Genes were grouped into functional groups using default settings and protocols.

#### 2.11.5 DMFS plot

Distant metastasis‐free survival (DMFS) plots were analyzed using the Kaplan–Meier plotting tool. Data (mRNA levels in triple–negative breast cancer subtype) were obtained from a recent database released in 2017 with 5,143 patients, and the Gene Set Analysis, Tumors module, was used. All gene expressions were equally weighted. To obtain clinical outcome according to each gene expression, we used the Sample Prediction module and classified the patients by the PAM classification method.

#### 2.11.6 Data accessibility

All ChIP–seq and AmpliSeq transcriptomic data have been deposited to Array Express under accession numbers E‐MTAB‐5241 and E‐MTAB‐5243, respectively.

## 3 RESULTS

### 3.1 Hs578T breast cancer cells have mesenchymal features, express high ZEB1 levels, and are sensitized to TGFβ signaling

**Figure 1:**
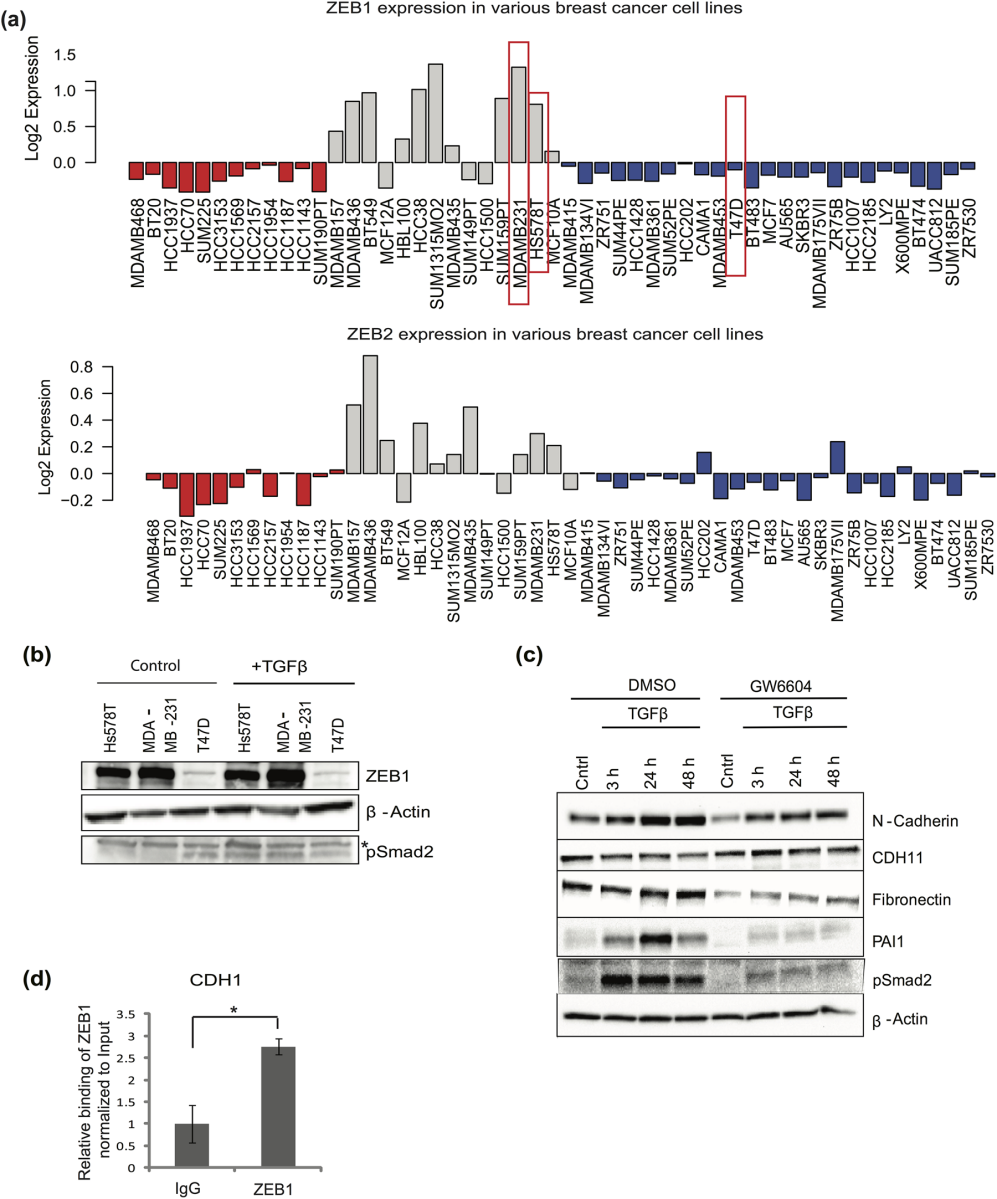
Hs578T cells have mesenchymal features, express high ZEB1 levels and are sensitized to TGFβ signaling. (a) Expression of *ZEB1* and *ZEB2* mRNA in different subtypes of breast cancer cells (basal‐A, red, basal‐B, gray, and Luminal, blue) based on expression values derived from the GOBO database. Red boxes indicate the cell lines with considerably high *ZEB1* mRNA expression, which were selected for further analysis. (b) Immunoblot showing expression of ZEB1 in Hs578T, MDA‐MB‐231, and T47D cells, after stimulation with or without TGFβ1 for 24 hr. β‐Actin serves as protein loading control and pSmad2 as a marker of TGFβ pathway activation; “*” indicates an unspecific protein band. (c) Immunoblot showing expression of epithelial and mesenchymal proteins in Hs578T cells in response to TGFβ1 stimulation for the indicated time periods in the absence (DMSO) or presence of GW6604 (5 µM) inhibitor. Cntrl indicates no stimulation with TGFβ1. β‐Actin serves as protein loading control and pSmad2 as a marker of TGFβ pathway activation. (d) Quantification of binding of ZEB1 to the *CDH1* promoter by ChIP–qPCR. An isotype–specific immunoglobulin control precipitation serves as reference. Statistical significance (*p*‐value <0.05) is shown based on a *t*‐test where *n* = 3

Expression screens have confirmed high constitutive expression of ZEB1 in many tumors (Gheldof, Hulpiau, van Roy, De Craene, & Berx, 2012). ZEB1 expression positively correlates with breast cancer aggressiveness and metastatic potential (Aigner et al., 2007; Spaderna et al., 2008). We confirmed this knowledge by querying the expression of *ZEB1* (and its related transcription factor *ZEB2*) in several human breast cancer cell lines based on data available in the GOBO database (Ringner et al., 2011). Cells with high *ZEB1* (and *ZEB2*) expression classified as basal‐B breast cancer cells (Figure 1a). Basal‐A and luminal epithelial breast cancer cells expressed low or undetectable levels of *ZEB1* (and *ZEB2*, Figure 1a). On the other hand, not all basal‐B cells exhibited robust mRNA levels for *ZEB1* (or *ZEB2*, Figure 1a). Basal‐B breast cancer cells are frequently reported as being EMT‐like tumor cells (Hennessy et al., 2009; Taube et al., 2010).

However, mRNA expression profiles, although useful and widely used, not always correlate with protein expression. We screened for ZEB1 protein expression among triple–negative breast cancer cells of the basal‐B subgroup, so that quantitative chromatin immunoprecipitation‐sequencing (ChIP–seq) analysis could be performed. We identified few such cell models. This protein expression screen, revealed that Hs578T and MDA‐MB‐231 cells, both of the basal‐B subgroup, express high levels of endogenous ZEB1 protein (Figure 1b), in agreement with the mRNA analysis (Figure 1a). In contrast, the luminal epithelial breast cancer cell line T47D exhibited essentially undetectable ZEB1 protein levels (Figure 1b), as expected based on the mRNA profile (Figure 1a). As a specificity control, we stimulated these cells with TGFβ, as this cytokine can induce EMT‐TF expression. However, induction of the already high ZEB1 protein levels by TGFβ could be occasionally observed but the effect was not convincing or easily quantifiable in these two basal‐B cell lines, Hs578T, and MDA‐MB‐231 (Figure 1b). The relative lack of response of ZEB1 to TGFβ also reflected the mesenchymal phenotype of Hs578T cells and the relative inability of TGFβ to induce further strong mesenchymal features. The mesenchymal proteins, N‐cadherin, CDH11 (cadherin‐11, also known as osteoblastic cadherin), and fibronectin, were either regulated or not at all regulated (e.g., CDH11) by TGFβ (Figure 1c). On the other hand, Hs578T cells were responsive to TGFβ, as phosphorylation of Smad2, and the immediate‐early target gene of TGFβ signaling, plasminogen activator inhibitor 1 (PAI1), responded robustly, and in a time‐dependent manner (Figure 1c). Furthermore, blocking the TGFβ receptor type I kinase using the chemical inhibitor GW6604 (Carthy, Engström, Heldin, & Moustakas, 2016), reduced the constitutive levels of N‐cadherin and fibronectin, supporting a dependence of their expression on autocrine TGFβ (Figure 1c). In the presence of GW6604, stimulation with TGFβ resulted in detectable time‐dependent induction of N‐cadherin, CDH11, fibronectin, and PAI1, confirming that Hs578T do not suffer from a defect in TGFβ signaling, but have been adapted to an autocrine TGFβ microenvironment and constitutive ZEB1 expression, that coordinately maintain the mesenchymal phenotype in these cells.

Finally, Hs578T cells generated reproducible and significant positive signals of ZEB1 association with the E‐cadherin (*CDH1*) promoter, upon ChIP analysis (Figure 1d). This experiment failed in most other basal‐B breast cancer models (data not shown) and for this reason Hs578T cells were selected for further analysis.

### 3.2 Genome–wide association of ZEB1 in triple–negative breast cancer cells

**Figure 2:**
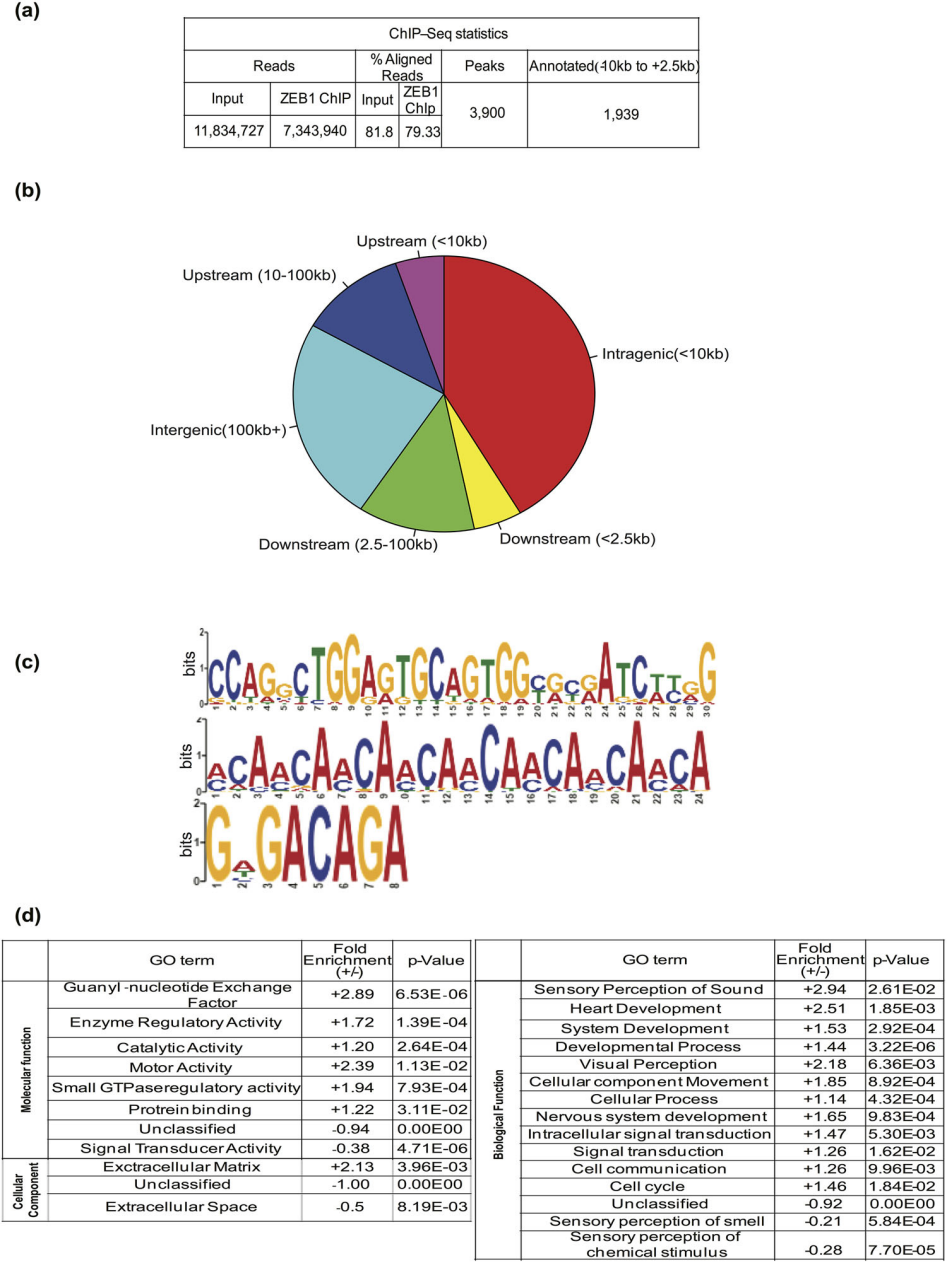
Genome–wide ZEB1 association in triple–negative breast cancer cells. (a) Table showing data and flow of analysis of the ChIP–Seq experiment from sequence reads to annotated gene assignment of the ZEB1 binding sites. (b) Pie chart showing the relative location of ZEB1 binding peaks on the Hg19 genome with respect to genes. (c) Visualization of statistically significant and centrally enriched DNA motifs, derived from the ZEB1 binding site sequences in Hs578T cells using the TOMTOM motif comparison tool. Sequence position is graphed on the x‐axis versus probability of frequency (bits) on the y‐axis. (d) Gene ontology (GO) analysis of annotated genes from the ZEB1 ChIP–Seq peaks into molecular function, biological process and cellular component categories using the GO Panther database. Relative fold‐enrichment and *p*‐values indicate significance of each functional category

We then performed high yield ChIP using the Hs578T cells and the same ZEB1 antibody used in Figure 1, followed by sequencing analysis (ChIP–Seq, Figure 2a, Supplementary Table S1). Almost 3,900 sequence peaks were characterized; the rate of alignment of the sequenced reads was steady and roughly 80% from all DNA samples, derived from input and immunoprecipitated chromatin (Figure 2a). Detailed analysis of the 3,900 peaks revealed intergenic regions (24% of total peaks), gene body regions, defined as sequences spanning from −100,000 to +100,000 bp relative to the transcription start site (TSS), which include intragenic (42% of total peaks) cis‐regulatory elements of transcriptional activity (Kowalczyk et al., 2012), upstream (<10,000 bp, 5.2% of total peaks), and downstream (<2,500 bp, 5.2% of total peaks) sequences (Figure 2b). Approximately 2,000 (52%) of these peaks of ZEB1 association to the genome mapped in regions proximal to the TSS, 10,000 bp upstream to 2,500 bp downstream of various genes (Figure 2a), and were studied in more detail.

Motif identification analysis of the genome–wide scale of ZEB1 binding sequences revealed that ZEB1 shares DNA binding regions with other transcription factors, such as RREB1, RUNX2 (Figure 2c first motif), and Smads (Figure 2c third motif). These motifs are centrally enriched, suggesting co‐regulation of target genes by ZEB1 and the above transcription factors. Additional DNA binding motifs were analyzed, some of which were centrally enriched with binding sites for transcription factors like ZNF354, EBF1, ESR1, FOXO1, and which were not centrally enriched and showed similar motifs with SRF, FOXI1, REST, Tcf3, Sox3, Spi1, or ELF5 (Supplementary Figure S1).

Gene ontology enrichment analysis revealed that ZEB1 associates with genes in diverse functional categories, including organ development, signal transduction, and cell communication, besides the EMT‐related genes (Figure 2d). Target genes in the class of motor activity, responsible for the catalysis of movement along microfilaments and microtubules, showed high fold (+2.39) enrichment (Figure 2d). The signal transduction class of genes showed negative enrichment, suggesting less functional relevance to ZEB1 (Figure 2d). In the super‐class of cellular components, ECM genes had a higher relevance, and fold enrichment (Figure 2d).

### 3.3 Novel ZEB1 targets in breast cancer cells

**Figure 3:**
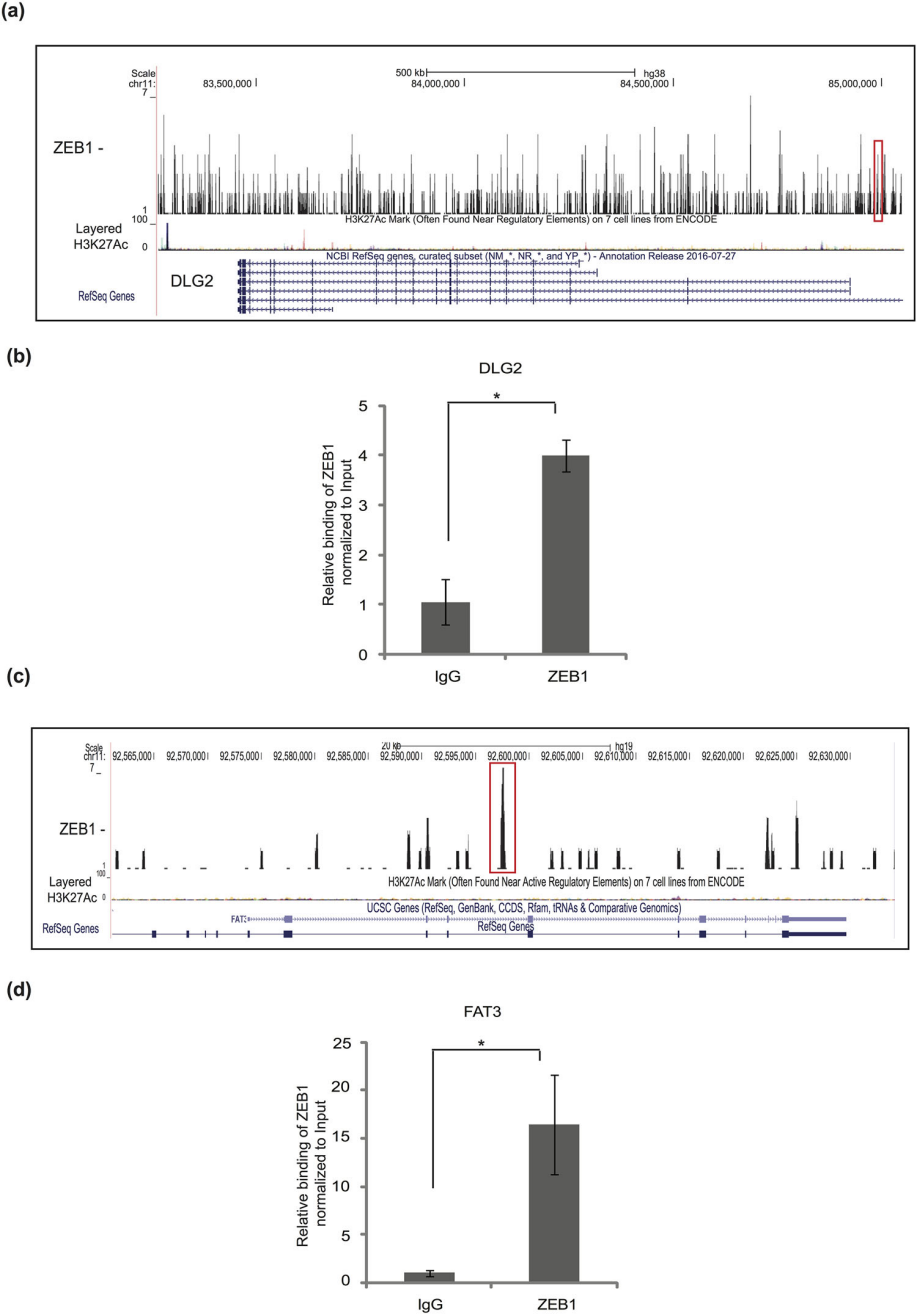
Novel gene targets of ZEB1 in breast cancer cells. (a–c) Representation of ZEB1 binding to the *DLG2* (a) and *FAT3* (c) genes; ChIP–Seq peaks (marked in red box) were aligned with tracks of H3K27Ac ChIP–Seq, which is used as a marker of gene activity, based on data available on the database, using the UCSC genome browser. (b–d) ChIP‐qPCR showing the significant enrichment of the *DLG2* (b) and *FAT3* (d) promoter regions in a ChIP experiment using the ZEB1 antibody, relative to the enrichment by non‐specific IgG. Statistical significance *p*‐value <0.002; *n* = 3

As part of a strict validation of peaks mapping between −10,000 to +2,500 bp from the TSS, we selected genes from two different classes of the GO analysis, nuclear binding (*DLG2*, *discs large MAGUK scaffold protein 2*) and embryo development (*FAT3*, *fat atypical cadherin 3*) (Figure 3). ChIP followed by quantitative qPCR, using as primers the DNA sequences corresponding to the coordinates of peaks obtained from the ChIP–Seq experiment, revealed significant ZEB1 binding to *DLG2* and *FAT3* (Figure 3b–d). Visualization of the ChIP–seq peaks on the UCSC genome browser alongside with the histone 3 lysine 27 acetylation pattern (H3K27Ac) commonly found in enhancer regions, showed that ZEB1 bound to enhancer‐like regions in these two genes (Figure 3a–c). A few additional genes were also validated for ZEB1 binding using ChIP‐qPCR (data not shown). We conclude that ChIP‐qPCR analysis can validate the genes identified by the genome–wide sequencing analysis.

### 3.4 ZEB1 knockout suppresses cell migration and anchorage‐independent growth

**Figure 4:**
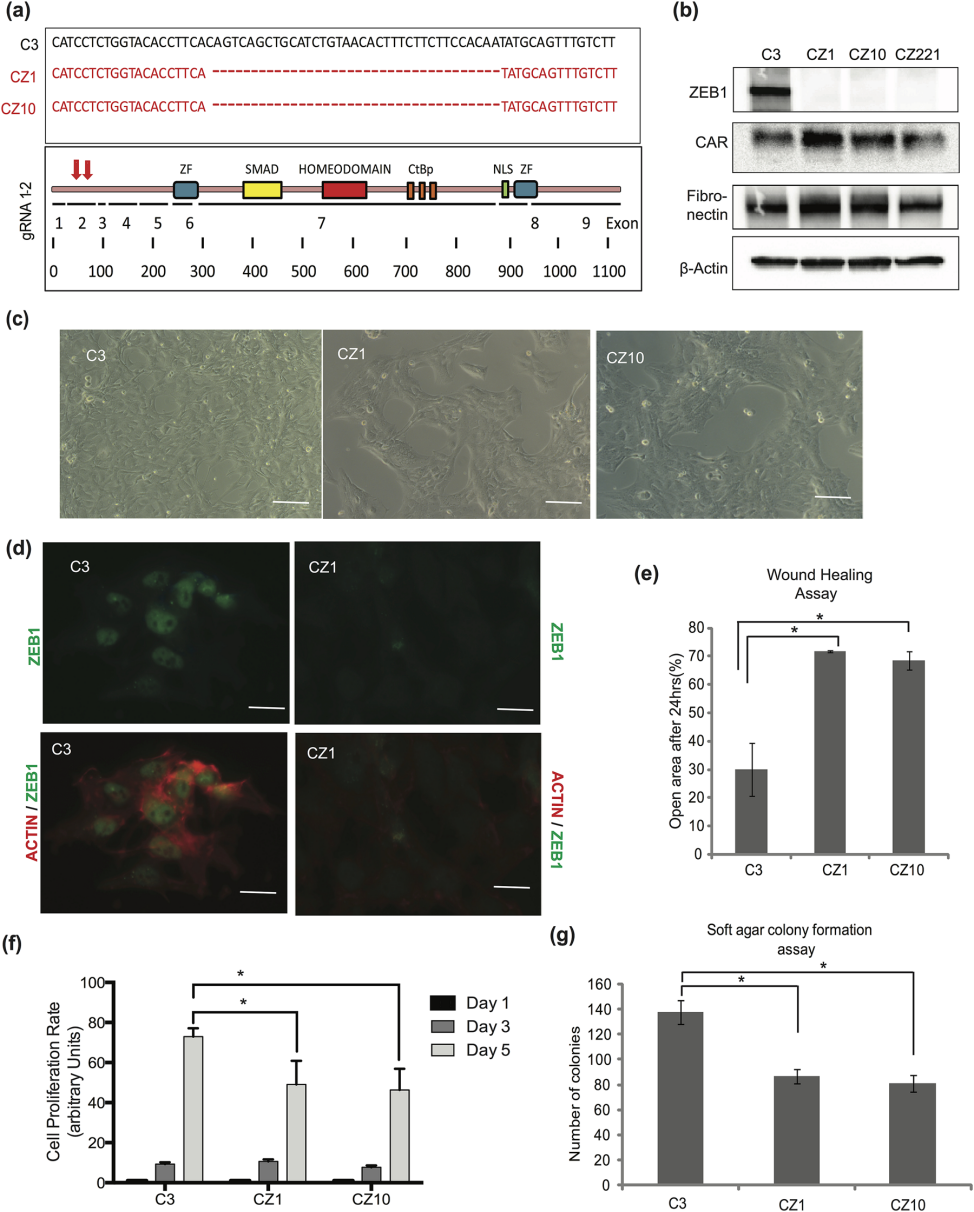
ZEB1 knockout suppresses cell migration and anchorage‐independent growth of Hs578T cells. (a) DNA sequences of the *ZEB1* gene in control cells C3 and deleted nucleotides in *ZEB1* knockout clones CZ1 and CZ10 using specific gRNA‐containing plasmids (red arrows on the ZEB1 cartoon). Diagram of the human ZEB1 protein with its functional domains, zinc fingers (ZF), Smad‐binding domain (SMAD), homeodomain, CtBp‐binding co‐repressor domain, and nuclear localization signal (NLS), along with the corresponding nine exons and numbering of the ZEB1 amino residues. (b) Immunoblot showing ZEB1 protein levels along with expression of the epithelial protein CAR and the mesenchymal protein fibronectin, and β‐actin that serves as protein loading control, in C3, CZ1, CZ10, and CZ221 Hs578T cell clones. (c) Phase contrast images showing cell morphology in C3, CZ1, and CZ10 Hs578T cell clones. Bars represent 50 μm. (d) Immunofluorescence microscopy images showing ZEB1 and polymerized actin in C3 and CZ1 Hs578T cell clones; ZEB1 alone (green) or ZEB1 merged with actin (red) images are shown. Bars represent 50 μm. (e) Quantification of migration assays by the T‐scratch software, showing % of open area 24 hr after a scratch was made in C3, CZ1, and C10 Hs578T cells. (f) Quantification of number of cells from C3, CZ1, and CZ10 Hs578T cells at days 1, 3, and 5 of cell growth after seeding. (g) Quantification of the number of colonies formed by the C3, CZ1, and CZ10 Hs578T cells in soft agar by the end of a 16‐day long incubation. Statistical significance *p*‐value <0.05; *n* = 3

As the germline knockout mouse of ZEB1 is lethal due to skeletal defects and severe T cell deficiency in the thymus (Takagi et al., 1998), and in order to exclude the chance of insufficient transient knockdown, a complete knockout was made in Hs578T cells using CRISPR‐CAS9 gene editing technology. The selected guideRNAs (gRNAs) targeted exon two of the gene body of *ZEB1* and the 2nd exon‐intron junction (Figure 4a). This resulted in a deletion of 30 bp (CZ1 and CZ10) when compared to control cells (C3) (Figure 4a). CRISPR‐CAS9 transfected cells were selected using puromycin‐resistance and were cloned prior to validation of the loss of ZEB1 expression, resulting in 18% success rate. Out of 13 ZEB1 knockout clones three representative clones were analyzed in more detail. Protein expression analysis showed a clear loss of ZEB1 protein expression in the clones CZ1, CZ10, and CZ221 (Figure 4b). ZEB1 is highly expressed in the Hs578T cells, and constitutive expression of ZEB1 acts as a repressor of epithelial and enhancer of mesenchymal genes. However, expression of the tight junction protein coxsackie and adenovirous receptor (CAR) (Raschperger et al., 2006) did not change in the CZ clones, which suggests ZEB1 knockout alone could not affect strongly expression of this epithelial protein (Figure 4b). Similarly, fibronectin, which is a mesenchymal protein marker, did not change in expression (Figure 4b), possibly reflecting a relative resistance of these cells to abandon their mesenchymal state and enhanced interaction of the cells with the ECM (Park & Schwarzbauer, 2014), as a putative compensatory mechanism in response to the loss of ZEB1.

Mesenchymal cells are distinguished from epithelial cells by distinct morphological features (Nieto et al., 2016). A morphological analysis of the cells has shown that CZ1 and CZ10 cells exhibited more cell‐cell junctions and appeared as less invasive compared to the C3 cells (Figure 4c). Anchorage‐independent growth apparent as cell detachment and growth on top of the monolayer was also observed in CZ1 and CZ10 clones compared to C3 cells (Figure 4c). Immunofluorescence analysis showed high expression of ZEB1 in the nucleus and high levels of filamentous actin in C3 cells, whereas CZ1 and CZ10 cells showed a clear loss of both ZEB1 and of organized filamentous actin. (Figure 4d). Examination of their migratory capacity showed that C3 cells were highly and significantly migratory, and CZ1 and CZ10 clones exhibited significant delay in migration during a T‐scratch assay (Figure 4e). Based on their morphological (Figure 4c) and growth (Figure 4f) characteristics, the cells were further analyzed by anchorage independent growth assays, which correlate to the oncogenic features of tumor cells. This assay further revealed that C3 cells formed larger (data not shown) and significantly higher number of colonies when cultured in soft agar for 16 days, when compared to CZ1 and CZ10 cells (Figure 4g). This pattern of growth correlated fully with the two‐dimensional rate of proliferation of these cells, whereby CZ1 and CZ10 cells failed to reach maximal growth rates (Figure 4f). Thus, inactivation of the *ZEB1* gene had significant phenotypic effects on these breast cancer cells.

### 3.5 Transcriptomic analysis of genes regulated by ZEB1

**Figure 5:**
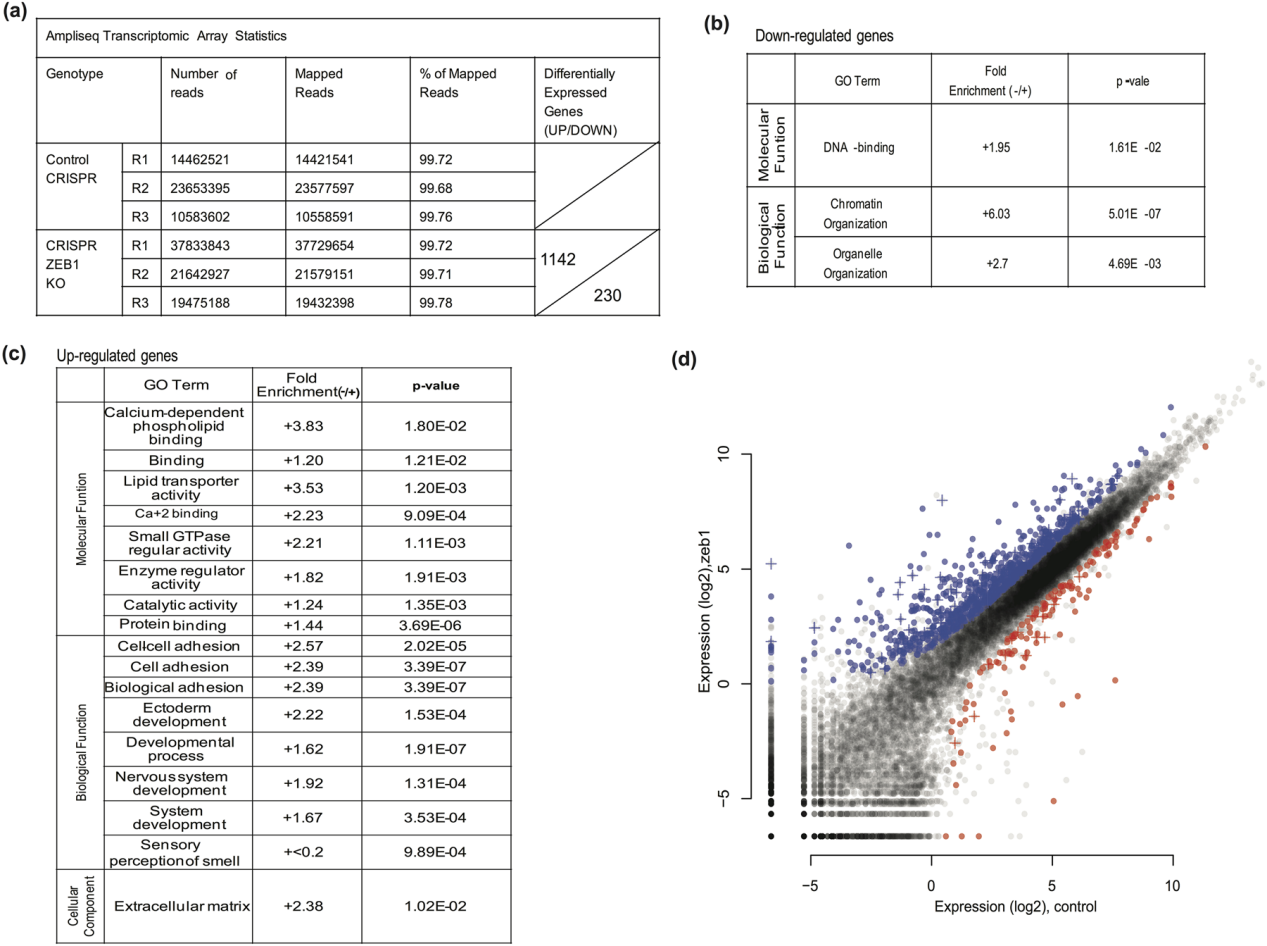
Transcriptomic analysis of genes regulated by ZEB1. (a) Table showing data and flow of analysis using AmpliSeq arrays measuring gene expression in triplicate biological replicates (R1‐R3) of C3 and CZ1 Hs578T cell clones, from number of reads obtained to number of differentially expressed genes (up‐regulated: top sector and down‐regulated: bottom sector) upon ZEB1 knockout. (b–c) Gene ontology (GO) analysis of annotated expressed genes, classified into molecular function, biological process, and cellular component categories using the GO Panther database. Relative fold‐enrichment and *p*‐values indicate significance of gene classification in each functional category. The data are divided into differentially expressed and significantly down‐regulated (b) or up‐regulated (c) genes upon ZEB1 knockout. (d) Profile of all the RefSeq genes based on their relative expression in the CZ1 Hs578T clone plotted against the C3 Hs578T clone. Up‐regulated (blue) and down‐regulated (red color) genes in the ZEB1 knockout clone along with statistically non‐significant expressed genes (gray color) are plotted; genes identified using the ChIP–Seq analysis (Figure 2) are superimposed (+ symbol)

The availability of a clean knockout system in the tumor cells, led us to perform a whole genome transcriptomic analysis, using the AmpliSeq assay in order to analyze the expression levels of all RefSeq genes in the C3 and CZ1 cells (Figure 5, Table S2). The assay uses targeted enrichment of over 21,000 genes and resulted in 10.5–37.8M reads out of which over 99% aligned to the human reference genome (Figure 5a). The expression profile of knockout cells (CZ1) when normalized with control Hs578T cells (C3) resulted in 1,142 (blue dots) and 230 (red dots) up‐regulated and down‐regulated genes respectively, with many genes expressed but having unchanged levels (gray dots) (Figure 5a–d). To our surprise, we found a substantial set of genes that were down‐regulated in ZEB1 knockout cells, which suggests that ZEB1 could act as a direct positive regulator of transcription; this class included DNA binding, chromatin organization, and organelle organization genes (Figure 4b). Gene Enrichment analysis showed that the positive transcriptional action of ZEB1 is compatible with previous analyses (Lehmann et al., 2016). On the other hand, and as expected, ZEB1 showed a negative correlation with a larger set of genes belonging to classes like lipid binding, protein binding, enzymatic activity, small molecule transportation, and calcium‐dependent phospholipid binding, which scored positive, with a 3.83‐fold enrichment relative to all RefSeq genes (Figure 5c). ZEB1 also exhibited a strong impact on the expression of genes linked to the regulation of cell adhesion, system development, and sensory development (Figure 5c).

To measure the direct impact of ZEB1 on gene expression, both ChIP–Seq and transcriptomic array data were over‐laid (Figure 5d, “ **+ **”), which resulted in a total of 154, 21 down‐regulated and 133 up‐regulated genes (Figure 5d, Supplementary Table S3). Gene ontology analysis on this subset of 154 genes did not show significant enrichment for any specific functional class of genes (data not shown), because of the relatively low number of genes analyzed. These data suggest that ZEB1 regulates several hundreds of genes, but only a subset (around 11% of these) are direct target genes to which ZEB1 binds in triple–negative breast cancer cells.

### 3.6 ZEB1 regulates the metalloproteinase inhibitor TIMP3

**Figure 6:**
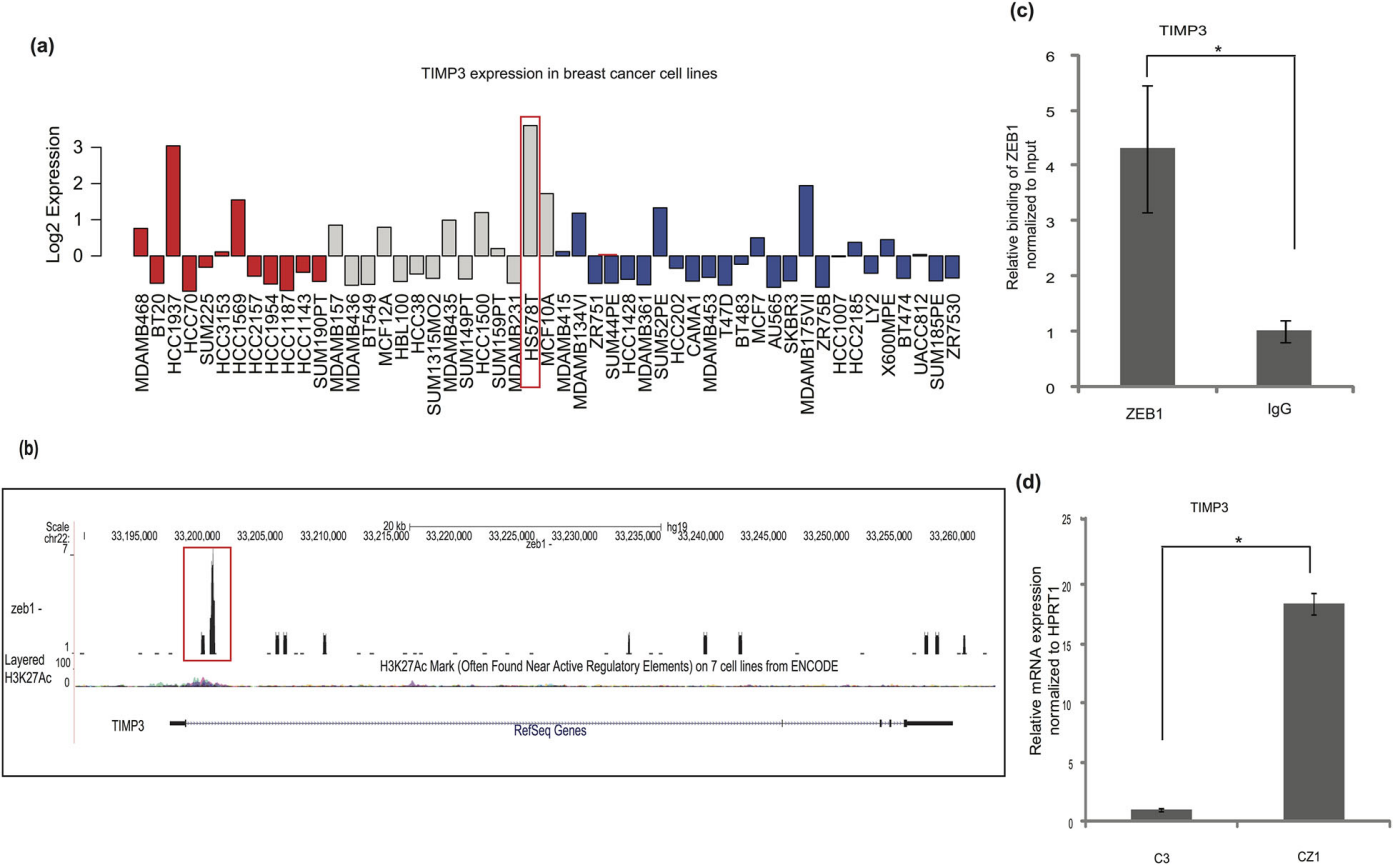
ZEB1 regulates the metalloproteinase inhibitor TIMP3. (a) Expression of *TIMP3* mRNA in different subtypes of breast cancer cell lines (basal‐A, red, basal‐B, gray, and Luminal, blue) based on expression values derived from the GOBO database. (b) Representation of ZEB1 binding to the *TIMP3* gene; ChIP–Seq peaks (marked in red box) and tracks of H3K27Ac ChIP–Seq, which is used as a marker of enhancer activity, using the UCSC genome browser. (c) ChIP–qPCR showing significant enrichment of the *TIMP3* promoter region in a ChIP experiment using the ZEB1 antibody, relative to enrichment by non‐specific IgG. (d) Relative amount of *TIMP3* mRNA expressed in C3 and CZ1 Hs578T cells after normalization with the *HPRT1* house‐keeping mRNA. Statistical significance *p*‐value <0.01; *n* = 3

One of the 133 up‐regulated genes in the ZEB1 knockout cells which exhibited binding of ZEB1 by ChIP–Seq analysis (Figure 5d) was tissue inhibitor of metalloproteinases 3 (TIMP3), a protein involved in the homeostasis of the ECM (Jackson, Defamie, Waterhouse, & Khokha, 2017). TIMP3 showed a varied level of expression in the breast cancer cells of the GOBO database (Figure 6a). In the basal‐A, basal‐B, and luminal subtypes, TIMP3 was expressed at high levels when the cells were more epithelial when compared to other cells; for example, *TIMP3* mRNA expression was higher in Hs578T when compared to MDA‐MB‐231 cells (Figure 6a). Genome–wide analysis of head and neck carcinomas showed that the *TIMP3* gene acquires DNA hypermethylation (Carvalho et al., 2008). The H3K27Ac enhancer marker profile showed that ZEB1 bound to a region of tandemly clustered enhancer elements (Figure 6b), which was validated by independent ChIP‐qPCR analysis (Figure 6c). In agreement with the AmpliSeq results, RT‐PCR analysis confirmed that *TIMP3* mRNA levels increased dramatically after ZEB1 knockout (Figure 6d). These data also support the notion that ZEB1 knockout cells drift toward an epithelial phenotype.

### 3.7 The transmembrane protein *TENM2* is a novel target gene of ZEB1

Another of the 133 up‐regulated genes in the ZEB1 knockout cells, which exhibited binding of ZEB1 by ChIP–Seq analysis (Figure 5d), was teneurin‐2 (TENM2) a cell adhesion transmembrane protein studied in neuronal and adipocyte progenitor cells (Tews et al., 2017). Gene ontology classifies TENM2 in the calcium ion binding and cell adhesion category, which was negatively regulated by ZEB1 (Figure 5c). On a global scale, transcriptomics and antibody‐based proteomics showed that TENM2 expression is higher in the heart and brain (Fagerberg et al., 2014), whereas breast tissue expression was not reported earlier.

**Figure 7:**
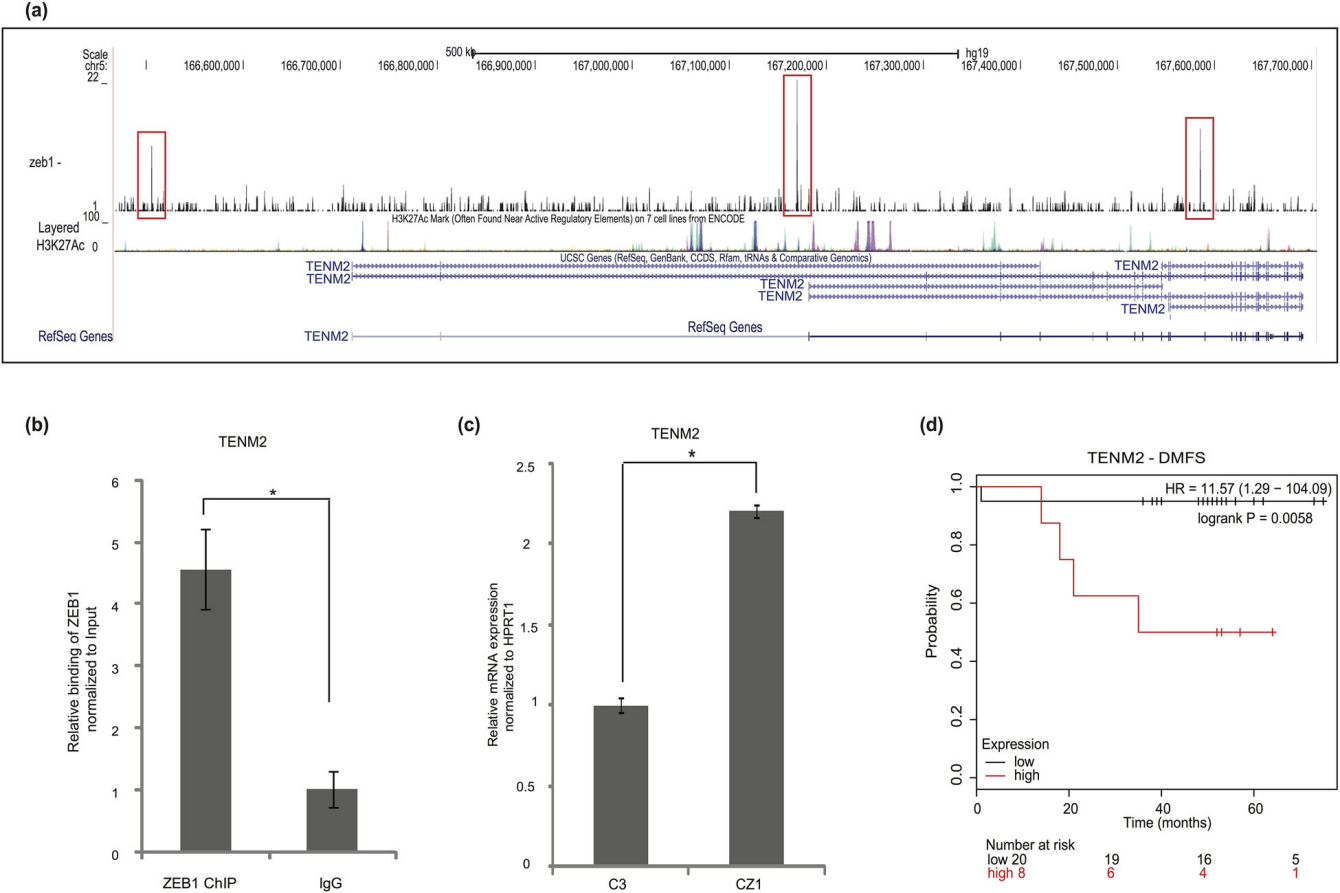
The transmembrane protein *TENM2* is a novel target gene of ZEB1. (a) Representation of ZEB1 binding to the *TENM2* gene; ChIP–Seq peaks (marked in red box) and tracks of H3K27Ac ChIP–Seq, which is used as a marker of enhancer activity, based on data available in the UCSC genome browser. (b) ChIP–qPCR showing significant enrichment of the *TENM2* promoter region in a ChIP experiment using the ZEB1 antibody, relative to enrichment by non‐specific IgG. (c) Measurement of relative amount of *TENM2* mRNA expressed in C3 and CZ1 Hs578T cells after normalization with the *HPRT1* house‐keeping mRNA. (d) Kaplan–Meier plot of DMFS of patients (*n* = 28) belonging to the triple‐negative breast cancer group, relative to the probability of survival. Significant difference is shown for two groups of patients classified based on high and low levels of *TENM2* mRNA expression obtained from GOBO and graphed using the Kaplan–Meier plotter tool. Statistical significance *p*‐value <0.01; *n* = 3

Upon knockout of ZEB1, *TENM2* mRNA expression increased in triple–negative breast cancer cells (Figure 7). The ZEB1 binding coordinates correlated with the H3K27Ac enhancer marker on the *TENM2* gene (Figure 7a), which was verified by ChIP‐qPCR of ZEB1 binding to the promoter region of *TENM2* (Figure 7b). Validation of the AmpliSeq transcriptomic assay confirmed the relative increase of *TENM2* levels in CZ1 compared to C3 cells (Figure 7c). Analysis of distant metastatic‐free survival (DMFS) of triple–negative breast cancer patient samples and levels of *TENM2,* revealed a poor prognostic index for patients with high *TENM2* expression (Figure 7d). We therefore conclude that *TENM2* may represent a gene whose transcriptional repression by ZEB1 may reflect an adaptation of breast cancer cells to an intermediate level of malignancy.

## 4 DISCUSSION

Previous investigations on the transcription factor ZEB1 analyzed its contribution to the process of EMT and identified specific genes, whose expression is regulated by ZEB1 (Gheldof et al., 2012). By analyzing triple–negative breast cancer cells we identified a genome–wide binding profile of ZEB1 on a few 1,000 genes. CRISPR‐Cas9‐mediated knockout of ZEB1 in the same cells resulted in the misregulation of a similarly large number of genes. This experimental approach has revealed: a) previously unrecognized cohorts of genes, whose expression is regulated by ZEB1 in breast cancer cells and b) the unexpected finding that ZEB1 contributes to the oncogenic potential of breast cancer cells, a function that may not necessarily link to the process of EMT.

Transcriptomic analyses across various carcinomas proposed association of *ZEB1* mRNA expression and tumor aggressiveness, including metastatic potential (Aigner et al., 2007; Spaderna et al., 2008; Taube et al., 2010). This is classically associated with the contribution ZEB1 makes to EMT (Gheldof et al., 2012). Our analysis of breast cancer cells agrees with this general conclusion, and associates high ZEB1 expression with the basal‐B subgroup of human breast cancers. Despite this clear picture derived from mRNA analyses, our effort to generate an equivalent result based on ZEB1 protein analysis across a panel of 15 human breast cancer cells, succeeded with only a small subset of these cell models (data not shown). This prohibited us from performing comparative ZEB1 ChIP–Seq analysis across several breast cancer models, aiming at understanding conservation and heterogeneity of ZEB1 binding in breast cancer populations. Most breast cancer cells that exhibit relatively high *ZEB1* mRNA, failed to present detectable ZEB1 protein based on direct immunoblotting, or ChIP followed by immunoblotting (data not shown). This points to post‐transcriptional mechanisms, including miRNAs of the *miR‐200* family (Burk et al., 2008), or of ubiquitin ligases, such as Siah (Chen et al., 2015) that keep ZEB1 protein levels low. Despite this difficulty, we analyzed Hs578T cells that express robust endogenous ZEB1 protein levels.

Hs578T cells belong to the triple–negative breast cancer group and classify under the basal‐B group and the claudin‐low subgroup that exhibits mesenchymal features (Hennessy et al., 2009; Taube et al., 2010). We confirmed this fact, and identified significant constitutive activity of TGFβ signaling in these cells. Reverting these cells to a more epithelial phenotype using a TGFβ receptor type I inhibitor (GW6604) was impossible. We conclude that Hs578T cells are mesenchymal and do not exhibit so‐called hybrid epithelial/mesenchymal (E/M) features, possibly reflecting a tumor cell type more enriched in cancer stem cells (Lambert et al., 2017).

ChIP–Seq analysis identified ∼3,900 genomic sites where ZEB1 binds and a large proportion of these sites mapped on (the TSS or within 10,000 bp or less) well characterized gene bodies. ZEB1 binds to the E‐box of *CDH1* and a few other genes (Gheldof et al., 2012). DNA binding motif identification analysis revealed that ZEB1 can additionally associate to DNA sequences, previously defined by other transcription factors, including RREB1, RUNX2, and Smad. ZEB1 binding to RUNX2 and Smad motifs in breast cancer cells appears functionally likely, as RUNX2 regulates mesenchymal progenitor to osteoblast differentiation, and mutations in RUNX2 perturb the RUNX2‐Smad association, linking to syndromes of craniofacial dysplasia (Zhang et al., 2000). Mice lacking ZEB1 also exhibit craniofacial malformations (Takagi et al., 1998), suggesting that ZEB1 and RUNX2 may act in synergy by regulating common genes during embryogenesis. The physical association between ZEB1 and Smad (Remacle et al., 1999) or between Smad and RUNX2 (Zhang et al., 2000), also underscore the importance of the motifs identified in this screen. The importance of a transcriptional crosstalk between ZEB1‐RUNX2 and Smad in breast cancer progression and EMT remains to be analyzed; such crosstalk most probably links to the mesenchymal program induced by these three transcriptional regulators.

The functional approach we used in order to validate ZEB1 target genes, *ZEB1* inactivation by CRISPR‐Cas9 targeting, generated some important conclusions. The combined ChIP–Seq and transcriptomic analysis generated a list of 154 genes whose levels changed after ZEB1 knockout and which exhibited direct binding of ZEB1 (Supplementary Table S3). These genes provide new clues to the function of ZEB1 in breast cancer. For example, *CDH1* and polarity genes (*Crumbs3*, *Lgl2*) are known direct target genes repressed by ZEB1 in carcinoma EMT (Aigner et al., 2007; Eger et al., 2005; Spaderna et al., 2008). We provide evidence for additional members of the extended cadherin family such as *FAT3* (Zhang et al., 2016), and polarity‐linked regulators such as *DLG2* (Roberts, Delury, & Marsh, 2012). Their functional relevance in breast cancer remains to be examined. Similarly, previous knowledge on the regulation of laminin‐332 (*LAMC2*) and integrin‐β4 (*ITGB4*) gene expression by ZEB1 (Drake et al., 2010), is now matched with new insight form our study that suggests that *TIMP3* and *TENM2* are downstream mediators of ZEB1 function. These specific genes are involved in ECM remodeling (*TIMP3*) and possibly in adhesion‐dependent regulation of cell differentiation (*TENM2*), which is worth examining further. In particular, TIMP3 can also be secreted to the ECM and has been established to induce mesenchymal or endothelial cell death, a mechanism that possibly depends on suppression of specific survival pathways (Koers‐Wunrau, Wehmeyer, Hillmann, Pap, & Dankbar, 2013; Qi & Anand‐Apte, 2015). Such pro‐apoptotic mechanism mediated by TIMP3 overexpression may explain the observed anti‐proliferative and reduced anchorage‐independent growth of the breast cancer cells where ZEB1 was eliminated, thus subsequently causing upregulation of endogenous TIMP3 expression. On the other hand, *TENM2* expression analysis across breast cancers provided interesting correlation with poor prognosis of patients that developed metastatic disease. Overall, the ZEB1 transcriptomic analysis provided more data for gene repression and less for gene activation. This is compatible with the current understanding of ZEB1 function, which positively regulates transcription when coupled to other signaling pathways, such as the Hippo/YAP (Lehmann et al., 2016).

The second unexpected finding with the ZEB1 knockout Hs578T cells was the suppression of oncogenicity observed in vitro in terms of anchorage‐independent growth. Some of the newly identified target genes of ZEB1 may thus be involved in survival and proliferation pathways, mechanisms possibly not directly linked to the EMT. This is compatible with ZEB1 being a pleiotropic transcription factor, and is supported by recent evidence that explains how ZEB1 contributes to tumor cell radioresistance by regulating homologous DNA recombination (Zhang et al., 2014). EMT is thought to be linked to a relatively slow cell proliferation, cell cycle arrest, and long‐term survival (Nieto et al., 2016). Accordingly, knockout of ZEB1 should have generated cells that proliferated faster or had enhanced anchorage‐independent growth. However, the result obtained was the opposite.

The third lesson derived from the *ZEB1* knockout analysis was the relative inability to revert the mesenchymal Hs578T breast cancer cells to epithelial cells. A trend toward epithelialization was obvious morphologically, but was not supported by molecular analysis of epithelial proteins, such as the tight junction protein CAR or E‐cadherin (data not shown). We conclude that removal of a single EMT‐TF, ZEB1, from an established mesenchymal breast cancer cell, is not sufficient to revert the tumor cells into a more epithelial phenotype, as we also demonstrated for Snail1 and Twist1 in an independent breast cancer model (Tan et al., 2015).

In summary, this work provides a useful resource for the exploration of novel functions of ZEB1 in the context of breast cancer. It opens the possibility of identifying ZEB1 target genes, which may allow interference with their function and generate, together with ZEB1 loss of function approaches, synthetic lethality that eliminates breast cancer cells.

## CONFLICTS OF INTEREST

The authors declare no conflicts of interest.

## AUTHORS’ CONTRIBUTION

Conception: AM. Design: VM and AM. Data acquisition: VM. Data analysis: VM, SE. Data interpretation: VM, SE, CHH, and AM. Article drafting and critical revision for important intellectual content: VM, SE, CHH, and AM. Final approval prior to publication: VM, SE, CHH, and AM.

## Supporting information


**Table S1**.

**Table S2**.

**Table S3**.

**Supporting Data S1**.
