# Supplementary material for: Genome–wide binding of transcription factor ZEB1 in triple‐negative breast cancer cells
Source: J Cell Physiol. 2018 May 10;233(10):7113–27. doi: 10.1002/jcp.26634 (PMC6055758; doi:10.1002/jcp.26634)
Supplement: Supplementary file 4 — Supporting Data S1. [file JCP-233-7113-s004.pdf]

Genome-wide binding of transcription factor ZEB1 in triple-negative breast cancer cells

Varun Maturi, Stefan Enroth, Carl-Henrik Heldin and Aristidis Moustakas

Supporting information

Supplementary Table S1. An Excel file including primary data from the ChIP-Seq analysis. This file tabulates in an organized manner the raw data deposited in Array Express under accession number E-MTAB-5241. The data are organized as: chromosome number, peak start, peak end, MACS, peak score, gene start, gene end, gene name, gene annotation, transcribed strand and distance from the gene start.

Supplementary Table S2. An Excel file presenting the primary data from the AmpliSeq analysis. This file tabulates in an organized manner the raw data deposited in Array Express under accession number E-MTAB-5243. The data are organized as: Gene name, Target ID, ENTREZ_GENE_ID, normalized expression values in triplicate samples of the mock C3 clone (up_242_1, up_242_2, up_242_3) and the ZEB1 KO CZ1 clone (up_242_7, up_242_8, up_242_9), mean of the mock C3 clone expression (meanCtrl), mean of the ZEB1 KO CZ1 clone expression (mean ZEB1), fold-change in expression between ZEB1 KO CZ1 and control C3 cells (absMeanFC), p-value (pval), adjusted p-value (padj), differentially expressed gene (DEG) and coincidence with the ChIP-Seq peaks (chipSeqPeak).

Supplementary Table S3. An Excel file presenting a short list of genes generated by the combination of ChIP-Seq and AmpliSeq analysis. The data are organized as: Gene name, mean expression values in the mock C3 clone (meanCntrl), mean expression values in the ZEB1 KO CZ1 clone (mean ZEB1 KO), fold-change in mean expression between ZEB1 KO CZ1 and control C3 cells (Mean fold-change), p-value (pval), adjusted p-value (padj), The top table (red) shows genes that are down-regulated when ZEB1 is lost, i.e. transcriptionally induced in the presence of ZEB1, and the bottom table (green) shows genes that are up-regulated when ZEB1 is lost, i.e. transcriptionally repressed in the presence of ZEB1.

Supplementary Figure S1. DNA motifs enriched in the ZEB1 ChIP-Seq experiment.
Visualization of statistically significant, centrally-enriched or not DNA motifs, derived from the ZEB1 binding site sequences in Hs578T cells using the TOMTOM motif comparison tool. Sequence position is graphed on the x-axis versus probability of frequency (bits) on the y-axis.

Predicted Motif of ZEB1 binding from ChIP-seq experiment	Central enrichment of Motif inside ChIP-seq Peak
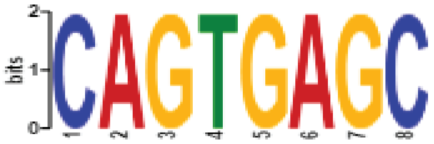	+
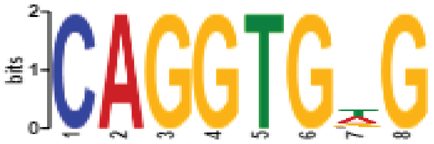	-
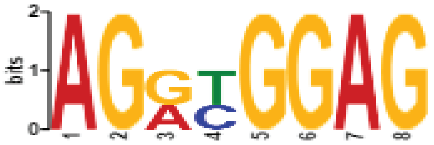	+
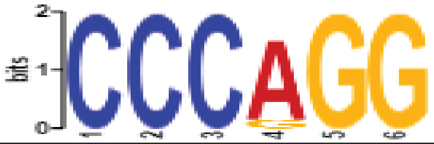	+
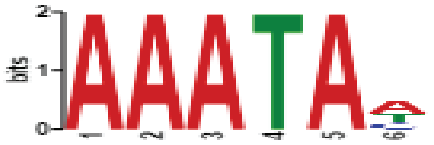	-
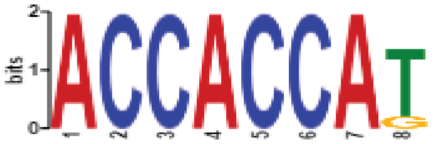	-
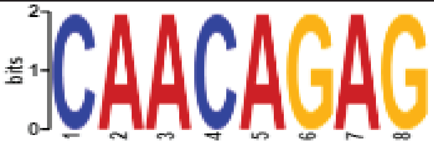	-
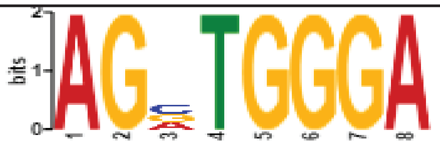	-
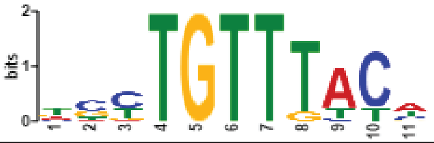	-
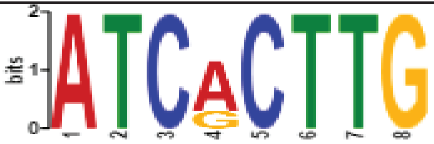	-
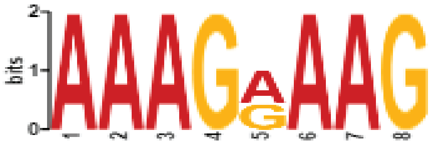	-

Maturi_Sup.Fig1.
